# Staphylococcus aureus Genomes Harbor Only MpsAB-Like Bicarbonate Transporter but Not Carbonic Anhydrase as Dissolved Inorganic Carbon Supply System

**DOI:** 10.1128/Spectrum.00970-21

**Published:** 2021-11-03

**Authors:** Sook-Ha Fan, Elisa Liberini, Friedrich Götz

**Affiliations:** a Microbial Genetics, Interfaculty Institute of Microbiology and Infection Medicine Tübingen (IMIT), University of Tübingen, Tübingen, Germany; University of Torino

**Keywords:** carbonic anhydrase, *Staphylococcus carnosus*, Firmicutes, MpsAB, *Staphylococcus aureus*, bicarbonate transporter

## Abstract

In recent years, it became apparent that not only autotrophic but also most other bacteria require CO_2_ or bicarbonate for growth. Two systems are available for the acquisition of dissolved inorganic carbon supply (DICS): the cytoplasmic localized carbonic anhydrase (CA) and the more recently described bicarbonate transporter MpsAB (membrane potential generating system). In the pathogenic species Staphylococcus aureus, there are contradictions in the literature regarding the presence of a CA or MpsAB. Here, we address these contradictions in detail. We could demonstrate by careful BLASTp analyses with 259 finished and 4,590 unfinished S. aureus genomes that S. aureus does not contain CA and that the bicarbonate transporter MpsAB is the only DICS system in this species. This finding is further supported by two further pieces of evidence: (i) *mpsAB* deletion mutants in four different S. aureus strains failed to grow under atmospheric air, which should not be the case if they possess CAs, since we have previously shown that both CA and MpsAB can substitute for each other, and (ii) S. aureus is completely resistant to CA inhibitors, whereas Staphylococcus carnosus, which has been shown to have only CA, was inhibited by ethoxyzolamide (EZA). Taken together, we demonstrate beyond doubt that the species S. aureus possesses only the bicarbonate transporter MpsAB as its sole DICS system.

**IMPORTANCE** The discrepancies in the current literature and even in NCBI database, which listed some protein sequences annotated as Staphylococcus aureus carbonic anhydrase (CA), are misleading. One of the existing problems in publicly available sequence databases is the presence of incorrectly annotated genes, especially if they originated from unfinished genomes. Here, we demonstrate that some of these unfinished genomes are of poor quality and should be interpreted with caution. In the present study, we aimed to address these discrepancies and correct the current literature about S. aureus CA, considering the medical relevance of S. aureus. If left unchecked, these misleading studies and wrongly annotated genes might lead to a continual propagation of wrong annotation and, consequently, wrong interpretations and wasted time. In addition, we also show that bicarbonate transporter MpsAB-harboring bacteria are resistant to CA inhibitor, suggesting that pathogens possessing both MpsAB and CA are not treatable with CA inhibitors.

## INTRODUCTION

Bicarbonate or hydrogen carbonate is a simple carbon molecule which occupies surprisingly crucial roles in various biological processes: for example, the tricarboxylic acid (TCA) cycle, cellular pH and volume regulation, and photosynthesis (1). The biochemistry of bicarbonate is fundamental to nearly all domains of life. For this reason, there are numerous pathways responsible for the fixation and assimilation of dissolved inorganic carbon (DIC), which consists mainly of free CO_2_ (gas), the bicarbonate ion (HCO_3_^−^), and carbonate ion (CO_3_^2−^) (2). In plants and most autotrophic bacteria, the first reaction of photosynthetic CO_2_ fixation is catalyzed by the enzyme ribulose-1,5-bisphosphate carboxylase/oxygenase (RuBisCO) via the Calvin-Benson-Bassham (CBB) cycle.

That being said, HCO_3_^−^ is also equally as important even for nonautotrophic bacteria due to the fact that many metabolic pathways require either HCO_3_^−^ or CO_2_ as the substrates or as products of metabolism (3, 4). In this regard, these bacteria utilize the enzyme carbonic anhydrase (CA) as a dissolved inorganic carbon supply (DICS) system (3, 5) and the more recently described bicarbonate transporter MpsAB (membrane potential generating system) in Staphylococcus aureus (4). MpsAB is present not only in autotrophic bacteria such as Hydrogenovibrio crunogenus, Nitrobacter winogradskyi (6), and Halothiobacillus neapolitanus (7) but also in many nonautotrophic bacteria, like some strains of Bacillus subtilis, Legionella pneumophila, and Vibrio cholerae (6, 8). MpsAB works alone and/or together with CA function to supply bicarbonate for anaplerotic reactions. Although both systems are interchangeable, they rarely coexist in a given species (4).

In our previous work, we showed that Staphylococcus carnosus harbors only a CA gene and confirmed that the protein is functional (8). As there was no S. carnosus-specific CA homolog present in S. aureus, we deduced that MpsAB functions as the sole CO_2_/bicarbonate concentration system in S. aureus. Moreover, MpsAB outperforms CA, and the former has an advantage in species where CO_2_ diffusion is impeded, for example in mucus biofilm-forming bacteria. As such, our findings are in contradiction with other studies about the presence of CA in S. aureus.

Since there are several publications in which an S. aureus-specific CA has been described and also studied (9–12), we investigated the question of whether a CA actually exists in S. aureus. Using BLASTp analyses, phenotypic characterization of *mpsAB* mutants, and the resistance studies to CA inhibitors, we demonstrate that there is no CA present in S. aureus.

## RESULTS

### All finished S. aureus genomes contain no CA-related Pfam motifs.

To enable a quick and systematic search for the presence of CA in S. aureus, we screened for the occurrence of protein families (Pfam) motifs (PFam00484, PFam00194, and PFam10563 for prokaryotic-type CAs, eukaryotic-type CAs, and putative CA-like domain, respectively) using the database from Integrated Microbial Genomes and Microbiomes (IMG/M) (13). We used this database instead of NCBI because it is more organized to perform searches and the exact strains from finished genomes, permanent drafts, or drafts could be selected. All 259 finished sequenced S. aureus strains do not contain any of the three CA-related Pfam motifs.

To demonstrate the reliability of Pfam motifs, we performed protein-protein Basic Local Alignment Search Tool search (BLASTp) of the protein sequence of an experimentally confirmed CA from Staphylococcus carnosus (8) against fully sequenced (finished) representative strains from the genus *Staphylococcus*. The presence of CAs based on Pfam motif correlated with the high percentage of protein identity from *S. carnosus* CA (Table 1). No significant protein identity was detected when there was no Pfam motif present, such as in S. aureus, Staphylococcus haemolyticus, and Staphylococcus lugdunensis. In addition, a search in all the 259 finished S. aureus genomes in IMG/G and also AureoWiki (14), which is manually curated, revealed that no protein is annotated as CA or putative CA.

**TABLE 1:** The presence of CAs inferred from Pfam motif correlates with the CA protein identity of *S. carnosus* in selected finished Staphylococcus genomes

Genome	CA^*a*^ (based on Pfam)		Identity (%) (amino acids aligned based on BLASTp)
	Pro	Euk	
Staphylococcus agnetis 908	+	−	73 (135/186)
Staphylococcus argenteus BN75	−	−	
Staphylococcus aureus *aureus* MSHR1132	−	−	
Staphylococcus aureus *aureus* USA300_FPR3757	−	−	
Staphylococcus capitis AYP1020	−	−	
Staphylococcus carnosus LTH 3730	+	−	100 (192/192)
Staphylococcus cohnii SNUDS-2	−	−	
Staphylococcus condimenti DSM 11674	+	−	97 (187/192)
Staphylococcus epidermidis RP62A	−	−	
Staphylococcus equorum KS1039	−	−	
Staphylococcus felis ATCC 49168	+	−	73 (135/186)
Staphylococcus haemolyticus JCSC1435	−	−	
Staphylococcus hominis *hominis* K1	−	−	
Staphylococcus hyicus ATCC 11249	+	−	72 (134/185)
Staphylococcus lugdunensis C_33	−	−	
Staphylococcus lutrae ATCC 700373	+	−	70 (133/191)
Staphylococcus muscae NCTC 13833	+	−	70 (130/188)
Staphylococcus nepalensis JS1	−	−	
Staphylococcus pasteuri SP1	−	−	
Staphylococcus pettenkoferi FDAARGOS_288	+	−	75 (140/192)
Staphylococcus piscifermentans NCTC 13836	+	−	96 (185/192)
Staphylococcus pseudintermedius ED99	+	−	70 (131/188)
Staphylococcus saprophyticus 883	−	−	
Staphylococcus schleiferi 1360-13	+	−	73 (132/185)
Staphylococcus sciuri SNUSD-18	+	−	67 (126/188)
Staphylococcus simiae NCTC 13838	−	−	
Staphylococcus simulans FDAARGOS_124	+	−	87 (167/192)
Staphylococcus stepanovicii NCTC 13839	+	−	66 (125/190)
Staphylococcus succinus 14BME20	−	−	
Staphylococcus warneri SG1	−	−	
Staphylococcus xylosus SMQ121	−	−	

a The presence of the proteins was inferred based on the following protein families (Pfam) domains search from finished bacterial genomes in the Integrated Microbial Genomes and Microbiomes (IGM/M) database: prokaryotic type-carbonic anhydrase (CA) (pro) (PFam00484), eukaryotic-type CA (euk) (PFam00194), and PFam10563 for putative CA-like domain. The symbols + and − indicate the presence or absence of the protein domains. Identity refers to identical residues shared with CA from *S. carnosus* (WP_015900702.1) using protein-protein Basic Local Alignment Search Tool (BLASTp).

### BLASTp showed no protein similarity of α-, β-, and γ-CAs in S. aureus.

Given that S. aureus and *S. carnosus* are from the same genus, they should share protein homology and more similarity with each other than with any bacteria from other genera. Therefore, the protein sequence of *S. carnosus* CA, which is from the class of β-CAs, was subjected to BLASTp search against all finished S. aureus genomes in IMG/M, but no similarity was found. As not all the microbial genomes might be integrated in IMG/M yet, we also performed the same BLASTp against S. aureus (taxonomy ID [taxid]: 1280) in NCBI database. We found two hits: NCBI accession numbers SPZ78436.1 and SPZ78435.1 (Table 2). As both the proteins are found in only one strain and based on the data in Table 2, most likely the genomes samples sequenced belonged to other staphylococcal species or the genes were wrongly annotated. Therefore, we concluded that there is no β-CA in S. aureus.

**TABLE 2:** Analysis of proteins wrongly annotated as S. aureus CA in NCBI^*a*^

NCBI accession no./length (amino acids)	Annotation in NCBI	Source (strain)	Comment
MBO8619751.1 (64)	Carbonic anhydrase family protein, partial (Staphylococcus aureus)	S. aureus strain IHMA68, unfinished genome with 268 contigs^*b*^	When this sequence was subjected to BLASTp search in NCBI, there was only one hit against its own sequence (100% identity). The rest of the hits were from multiple organisms with the highest identity from one Homo sapiens and other primates such as Hylobates moloch, Pan troglodytes, and Pongo abelii (86–91% identity).
MBO8666615.1 (77)	Carbonic anhydrase family protein, partial (Staphylococcus aureus)	S. aureus strain IHMA56, unfinished genome with 680 contigs^*b*^	When this sequence was subjected to BLASTp search in NCBI, there was only one hit against its own sequence (100% identity). The rest of the hits were from multiple organisms with the highest identity from Homo sapiens (100% identity, 100% protein coverage).
MVW54107.1 (151)	Carbonic anhydrase, partial (Staphylococcus aureus)	S. aureus strain mecC 165 PE, unfinished genome with 37 contigs	When this sequence was subjected to BLASTp search in NCBI, there was only one hit against its own sequence (100% identity). The rest of the 99 hits were from different bacteria like *Acidobacteria bacterium*, *Ignavibacteriales bacterium*, etc., with 47–91% identity (98–100% protein coverage).
NGB42162.1 (184)	Gamma-carbonic anhydrase family protein (Staphylococcus aureus)	S. aureus strain UG302, unfinished genome with 167 contigs^*c*^	When this sequence was subjected to BLASTp search in NCBI, there was no hit even against its own sequence or any S. aureus proteins. All of the 100 hits were from Salmonella enterica, with 99–100% identity.
NGG14433.1 (97)	Gamma-carbonic anhydrase family protein, partial (Staphylococcus aureus)	S. aureus strain UG271, unfinished genome with 397 contigs^*c*^	When this sequence was subjected to BLASTp search in NCBI, there was only one hit against its own sequence (100% identity). The rest of the 99 hits were all from Salmonella enterica with 99% identity and 100% protein coverage.
OWU61334.1	Carbonic anhydrase, partial (Staphylococcus aureus)	S. aureus strain W1, unfinished genome with 380 contigs	When this sequence was subjected to BLASTp search in NCBI, there was only one hit with 100% identity but it was annotated as SulP family inorganic anion transporter, partial from S. aureus. The rest were almost all from Mycobacterium tuberculosis (100% identity).
SPZ78435.1 (61)	Carbonic anhydrase (Staphylococcus aureus)	S. aureus strain NCTC12981, unfinished genome with 15 contigs	When this sequence was subjected to BLASTp search in NCBI, there was only one hit against its own sequence (100% identity). The rest were multiple hits from other Staphylococcus species, with the highest identity from *Staphylooccus coagulans* (98% identity with 86% coverage).
SPZ78436.1 (193)	Carbonic anhydrase (Staphylococcus aureus)	S. aureus strain NCTC12981, unfinished genome with 15 contigs	When this sequence was subjected to BLASTp search in NCBI, there was only one hit against its own sequence (100% identity). The rest were multiple hits from other Staphylococcus species with the highest identity from Staphylococcus schleiferi which covers 69% of the protein length with 100% identity
WP_094666538.1 (149)	Gamma-carbonic anhydrase family protein, partial (Staphylococcus aureus)	S. aureus strain UV695, unfinished genome with 468 contigs	When this sequence was subjected to BLASTp search in NCBI, there was only one hit against its own sequence (100% identity) and almost all the hits were from Enterococcus faecium or *Enterococcus* sp. with 99–100% identity.

a SulP, sulfate permease.

b Submitted by the same group.

c Submitted by the same group.

Since the different classes of CAs have independent evolutionary origins (3), we also searched for the presence of α- and γ-CAs in S. aureus. We selected some bacteria whose CAs were experimentally proven, and these protein sequences were subjected to BLASTp search in S. aureus, as well as two CA-harboring species, *S. carnosus* and Staphylococcus pseudintermedius, as controls (Table 3 and 4). As shown in Table 3, there was no similarity among these CAs with S. aureus, *S. carnosus*, and S. pseudintermedius except for two cases. First, BLASTp of two human CAs resulted in two hits with proteins annotated as S. aureus CA (Table 3). Considering that they are found in only two unfinished S. aureus genomes (Table 3) and the errors observed in these sequences (Table 2), the genome samples were most likely contaminated. For the same reason as that mentioned previously, we deduced that there are no α- and γ-CAs in S. aureus (Table 3 and 4).

**TABLE 3:** Protein sequence similarity search for selected α-CAs in the genomes of S. aureus, *S. carnosu*s, and S. pseudintermedius using BLASTp

Species	UniProt ID/length (amino acids)	Ref	Identity (%)^*a*^/protein coverage (amino acids)			Comment
			S. aureus (NCBI taxid: 1280)	*S. carnosus* (NCBI taxid: 1281)	S. pseudintermedius (NCBI taxid: 283734)	
Enterococcus faecium	Q3XYE8 (234)	33	No significant similarity found	No significant similarity found	No significant similarity found	
Helicobacter pylori	A0A0M3KL20 (234)	34	No significant similarity found	No significant similarity found	98 (53/54) (WP_181892146.1) in only one unfinished genome of S. pseudintermedius strain ST525	When WP_181892146.1 was subjected to BLASTp search in NCBI BLASTp, it showed only one hit against its own sequence and the rest of the 99 hits were from H. pylori with 98–100% identity.
*Neisseria gonorrhoaea*	Q50940 (252)	35, 36	No significant similarity found	No significant similarity found	No significant similarity found	
Vibrio cholerae	Q9KMP6 (239)	37	No significant similarity found	No significant similarity found	37 (18/49) (WP_181892146.1) in only one unfinished genome of S. pseudintermedius strain ST525	Same comment as in H. pylori
Human CA1	P00915 (261)	38, 39	38 (27/72) (MBO8666615.1) in only one unfinished genome of S. aureus strain IHMA56 and another hit of 37 (22/60) (MBO8619751.1) in only one unfinished genome of S. aureus strain IHMA68	No significant similarity found	No significant similarity found	See Table 2 for comment on this protein
Human CA2	P00918 (260)	38, 40	50 (27/54) (MBO8619751.1) in one unfinished genome of S. aureus strain IHMA68 and another hit 37 (24/75) (MBO8666615.1) in one unfinished genome of S. aureus strain IHMA56	No significant similarity found	No significant similarity found	See Table 2 for comment on this protein

a Identity refers to shared identical residues with each of the CA proteins (UniProt ID) from selected bacteria and the indicated Staphylococcus species using BLASTp.

**TABLE 4:** Protein sequence similarity search for selected γ-CAs in the genomes of S. aureus, *S. carnosus*, and S. pseudintermedius using BLASTp

Species	UniProt ID/length (amino acids)	Ref	Identity (%)^*a*^/protein coverage (amino acids)		
			S. aureus (NCBI taxid: 1280)	*S. carnosus* (NCBI taxid: 1281)	S. pseudintermedius (NCBI taxid: 283734)
Enterococcus faecium	Q3XX77 (161)	33	3 hits in S. aureus annotated as γ-CAs (NGG14433.1, NGB42162.1, and WP_094666538.1)^*b*^ and the rest of the hits are from S. aureus proteins annotated as phenylacetic acid degradation protein PaaY with low identity (39, 32/83) and/or sugar O-acetyltransferase	No significant similarity found	No significant similarity found
Escherichia coli	P0A9W9 (184)	41, 42	3 hits in S. aureus annotated as γ-CAs (NGG14433.1, NGB42162.1, and WP_094666538.1)^*b*^ and another as phenylacetic acid degradation protein PaaY in S. aureus	No significant similarity found	No significant similarity found
Methanosarcina thermophila	P40881 (247)	43	3 hits in S. aureus annotated as γ-CAs (NGG14433.1, NGB42162.1, and WP_094666538.1)^*b*^	No significant similarity found	No significant similarity found
Halobacterium salinarum	Q9HR64 (220)	44	31 (51/163) (MVW54107.1)^*b*^ in only one unfinished genome of S. aureus strain mecC 165 PE	No significant similarity found	No significant similarity found

a Identity refers to shared identical residues with each of the carbonic anhydrase (CA) protein (UniProt ID) from selected bacteria and the indicated Staphylococcus species using BLASTp.

b See Table 5 for comments regarding these protein sequences.

### BLASTp of all proteins annotated as S. aureus CAs revealed errors in permanent draft genomes.

To further confirm that there is indeed no CA present in S. aureus, we searched the NCBI database for all the proteins annotated as S. aureus CA and performed an extensive BLASTp search. All 259 finished genomes showed no identity at all against the seven CAs listed in Table 5, except for two cases where low identities were found in in proteins annotated as acetyltransferase, galactoside O-acetyltransferase, sulfate permease, or hypothetical protein but not as CA. As with the BLASTp of α-, β-, and γ-CAs above, almost all of the CA similarities found were in unfinished genomes, suggesting that these genomes are often unreliable and should be interpreted with caution. To prove our point, we extended the same BLASTp search in 4,590 unfinished genomes. Results similar to those found with finished genomes were found, and all other hits were found in assemblies which were marked as contaminated by NCBI (Table 5). One particular strain, C0673, showed multiple hits for NGG14433.1, NGB42162.1, SPZ78435.1, SPZ78436.1, and WP_094666538.1.

**TABLE 5:** Homology of protein annotated as S. aureus CAs in all finished and permanent genomes sequences of S. aureus^*a*^

Accession no./protein length (amino acids)	Identity (%)^*b*^/protein coverage (amino acids)	Comment
	Finished genomes in IMG/M (259 strains)	
MBO8619751.1 (64)	No significant similarity found	
MBO8666615.1 (77)	No significant similarity found	
MVW54107.1 (151)	No significant similarity found	
NGB42162.1 (184)	No significant similarity found	
NGG14433.1 (97)	No significant similarity found	
OWU61334.1 (172)	No identity in 253 strains, except in 5 strains which showed 27 (46/170) identity in proteins annotated as sulfate permease	
SPZ78435.1 (61)	No significant similarity found	
SPZ78436.1 (193)	No significant similarity found	
WP_094666538.1 (149)	All strains show 33 (40/122) identity in proteins annotated as acetyltransferase (isoleucine patch superfamily), acetyltransferase-like (isoleucine patch superfamily), galactoside O-acetyltransferase, or hypothetical protein	Annotated as such because these sequences have the related COG, KOG, or Pfam motifs
	Permanent draft genomes in IMG/M (4,590 strains)	
MBO8619751.1 (64)	No significant similarity found	
MBO8666615.1 (77)	No significant similarity found	
MVW54107.1 (151)	No identity in all 4,586 strains except 4 strains: DEU37 (30 [49/163] as CA, partial gene [“no stop”]), DEU28 (30 [49/163] as CA), DEU35 (33 [39/117] as CA, partial gene [“no start”]), DEU41 (33 [39/117] as CA, partial gene [“no start”])	All the 4 strains listed here were marked as “anomalous assembly: contaminated” by NCBI
NGB42162.1 (184)	No identity in 4,584 strains except DEU28, DEU35, DEU37, DEU41, and DEU42 (37 [57/156] as CA or acetyltransferase), DEU39 (36% [31/86] as transferase hexapeptide [six repeat-containing protein]), C0673 (39 [67/171] as CA or acetyltransferase encoded by gene V070_00826)	All the 6 strains listed here were marked as “anomalous assembly: contaminated” by NCBI. C0673 is wrongly annotated as S. aureus in NCBI database
NGG14433.1 (97)	No identity in all 4,583 strains except DEU28, DEU35, DEU37, DEU41, and DEU42 (35 [27/77] as CA or acetyltransferase), DEU39 (partial gene [“no start”]), C0673 (41 [38/92] as CA or acetyltransferase encoded by gene V070_00826)	All the 6 strains listed here were marked as “anomalous assembly: contaminated” by NCBI. C0673 is wrongly annotated as S. aureus in NCBI database
OWU61334.1 (172)	No identity in 4,514 strains except in 76 strains which showed 27 (46/170) identity in proteins annotated as sulfate permease	
SPZ78435.1 (61)	No identity in all 4,589 strains except C0673 (66 [35/53] as CA encoded by gene V070_02709)	C0673 is wrongly annotated as S. aureus in NCBI database
SPZ78436.1 (193)	No identity in all 4,589 strains except C0673 (71 [95/133] as CA encoded by gene V070_02709 and another 25 [32/126] as CA encoded by V070_01492)	C0673 is wrongly annotated as S. aureus in NCBI database
WP_094666538.1 (149)	All 4,590 strains showed 33 (40/122) as acetyltransferase (isoleucine patch superfamily), galactoside O-transferase, or hypothetical protein. A few strains have unspecific hits, for example: NRS384 (35 [33/95] as hexapeptide repeat of succinyltransferase), OCMM6067 (32 [23/66] as 2,3,4,5-tetrahydropyridine-2-6, dicarboxylate *N*-acetyltransferase), 65-1322 (33 [40/122] as transferase hexapeptide repeat-containing protein), ATCC BAA-39 (33 [40/122] as galactoside-6- phosphate isomerase LacA subunit), C0673 (50 [72/144] as CA or acetyltransferase encoded by a gene V070_00826, 35 [26/77] as maltose O-acetyltransferase in gene V070_00366, and 25 [26/103] as acetyltransferase [isoleucine patch superfamily] encoded by gene V070_00906).	These strains have the same similarity as all the finished genomes. As there only a few strains out of 4,590 permanent draft sequences with unspecific and low identity, the origins of each of these strains were not examined. C0673 is wrongly annotated as S. aureus in NCBI database.

a COG, Clusters of Orthologous Genes; KOG, Eukaryotic Orthologous Groups; IGM/M, Integrated Microbial Genomes and Microbiomes; Pfam, protein families.

b Identity refers to shared identical residues with each of the CA proteins (NCBI accession number) against the indicated S. aureus genomes in IMG/M database using BLASTp.

According to NCBI, C0673 is an unfinished genome with 89 contigs where the taxonomy check is inconclusive. Although this strain is annotated as S. aureus C0673 in NCBI database, it is highly questionable. Thus, we downloaded the genome sequence and checked it against public databases for molecular typing and microbial genome diversity (PubMLST) (15). According to PubMLST, the predicted taxa for C0673 are actually 83% Staphylococcus sciuri, now known as Mammaliicoccus sciuri. Using the IMG/M database, pairwise average nucleotide identity (ANI) with two finished S. sciuri genomes revealed that C0673 has 97% nucleotide identity with S. sciuri SNUDS-18 and 96% nucleotide identity with S. sciuri FDAARGOS_285. C0673 is wrongly annotated as S. aureus in NCBI database, which gave us false-positive hits in our BLASTp because S. sciuri but not S. aureus has a CA as stated in Table 1 and our previous work (8).

### All protein sequences annotated as S. aureus CAs in NCBI are not from S. aureus.

Given the observation that not a single strain out of 4,849 S. aureus genomes has a reasonable protein identity with any of the sequences annotated as S. aureus CA, we proceeded to examine the authenticity of these sequences. All of the nine sequences listed in Table 2 originated from unfinished genomes, and most of them contain many contigs, indicating these genomes are of low quality (16). When these sequences were subjected to BLASTp search, they showed only one hit against their own sequences and the rest were from either other staphylococcal species or other microorganisms, or even human. This clearly suggests that these genome assemblies were contaminated or contain sequencing errors and therefore are not accurate and should be corrected.

### Deletion of *mpsAB* in four different backgrounds of S. aureus causes severe growth defect in atmospheric conditions.

In our previous study, we demonstrated that deletion of *mpsAB* in two different S. aureus backgrounds, SA113 and HG001 (both are methicillin-susceptible S. aureus), could not grow under normal atmospheric conditions, indicating that there is no functional CA (4, 17). Here, we deleted *mpsAB* in two more S. aureus strains, JE2 and MW2, which are methicillin resistant (MRSA). Like with SA113 and HG001, the MRSA deletion mutants could not grow under atmospheric air, indicating that MpsAB is the only DICS system (Fig. S1).

### MpsAB-harboring strains are resistant to CA inhibitors.

CA inhibitors, especially sulfonamides, are able to effectively inhibit most of the CAs and consequently hinder the bacterial growth (18, 19). With regard to this, we tested eight such inhibitors to provide further evidence that CA does not present in S. aureus. Acetazolamide (AZA), ethoxyzolamide (EZA), dorzolamide (DOR), and methazolamide (MEZ) are FDA-approved CA inhibitors used in the treatment of glaucoma, while celecoxib (CEL), chlorthalidone (CL), and famotidine (FAM) are a nonsteroidal anti-inflammatory agent, a thiazide diuretic, and an antiulcer drug, respectively (20–22). S0859 is an *N*-cyanosulphonamide synthetic compound reported to be a selective inhibitor of sodium-bicarbonate cotransporters (NBC, SLC4) in mammalian heart (23). The chemical structures are provided in Figure S2.

**FIG 1:**
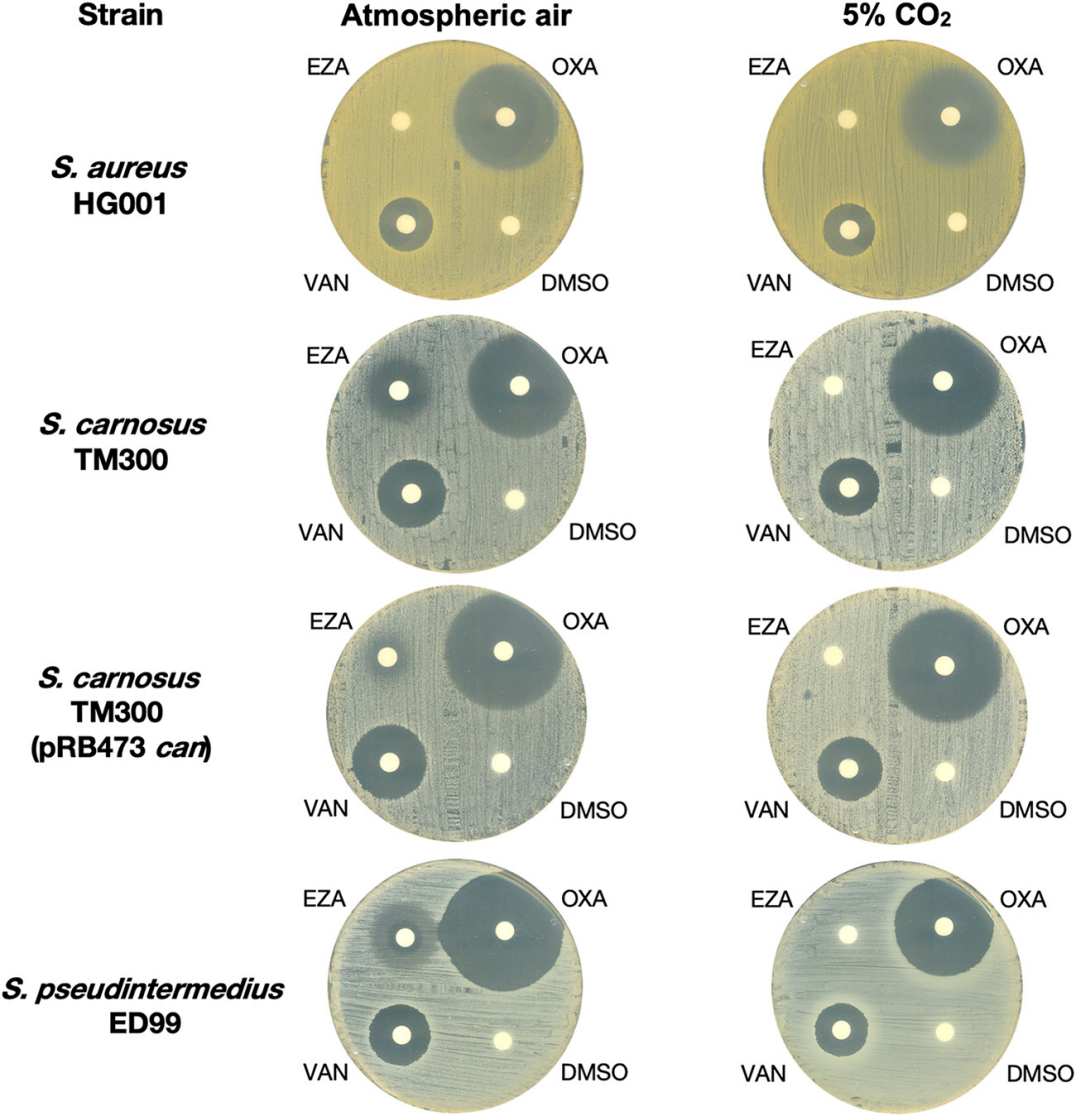
Disk diffusion results showing inhibition zones of CA inhibitor ethoxyzolamide (EZA) against selected staphylococcal strains. The Mueller-Hinton agar plates were inoculated with S. aureus HG001, *S. carnosus* TM300, and *S. carnosus* TM300 (pRB473-can), in which the CA was overexpressed, and S. pseudintermedius ED99. Paper disks impregnated with 10 μl of EZA, oxacillin (OXA), and vancomycin (VAN) as positive controls at concentrations of 1 mM each and appropriate concentration of DMSO as negative control were incubated at 37°C overnight in atmospheric and CO_2_ conditions.

At the highest concentration tested (1,000 μM), all the compounds did not inhibit MpsAB-harboring S. aureus and S. epidermidis as well as strains where CAs were deleted and complemented with MpsAB instead, including *S. carnosus* carrying plasmid containing *mpsABC* (Table 6). In CA-harboring strains, only EZA showed an MIC of 64 μM against *S. carnosus*, which was increased to 250 μM when CA was overexpressed in *S. carnosus* TM300 (pRB473 *can*) (Table 6). To verify that activity of EZA is mediated through the inhibition of CA, we repeated the MIC determinations in both normal atmospheric air and 5% CO_2_ conditions. EZA was inactive against these strains when incubated in the presence of 5% CO_2_ compared to atmospheric air, while there was no difference in S. aureus (Fig. 1; Table S3). Vancomycin and oxacillin were used as a control and, as expected, displayed no difference in MIC in both conditions. Collectively, these results suggest that the target for EZA is most likely the intracellular CA, which is not present in S. aureus and S. epidermidis.

**TABLE 6:** MIC values of CA inhibitors against selected staphylococcal strains^*a*^

Dissolved inorganic carbon supply (DICS) system	Strain	MIC (μM)^*b*^							
		AZA	EZA	DOZ	MEZ	CT	FAM	S0859	CEL
MpsAB	S. aureus HG001	>1,000	>1,000	>1,000	>1,000	>1,000	>1,000	>1,000	>1,000
	S. epidermidis O47	>1,000	>1,000	>1,000	>1,000	>1,000	>1,000	>1,000	>1,000
	*S. carnosus* TM300 Δ*can* (pRB473 *mpsABC*)	>1,000	>1,000	>1,000	>1,000	>1,000	>1,000	>1,000	>1,000
	*S. carnosus* TM300 (pRB473 *mpsABC*)	>1,000	>1,000	>1,000	>1,000	>1,000	>1,000	>1,000	>1,000
	S. pseudintermedius ED99 Δ*can* (pRB473 *mpsABC*)	>1,000	>1,000	>1,000	>1,000	>1,000	>1,000	>1,000	>1,000
CA	*S. carnosus* TM300	>1,000	64	>1,000	>1,000	>1,000	>1,000	>1,000	>1,000
	*S. carnosus* TM300 (pRB473 *can*)	>1,000	250	>1,000	>1,000	>1,000	>1,000	>1,000	>1,000
	S. pseudintermedius ED99	>1,000	250	>1,000	>1,000	>1,000	>1,000	>1,000	>1,000
	S. aureus HG001 (pRB473 *can*)	>1,000	>1,000	>1,000	>1,000	>1,000	>1,000	>1,000	>1,000
	S. aureus HG001 Δ*mpsABC* (pRB473 *can*)	>1,000	>1,000	>1,000	>1,000	>1,000	>1,000	>1,000	>1,000

a AZA, acetazolamide; EZA, ethoxyzolamide; DOR, dorzolamide; MEZ, methazolamide; CT, chlorthalidone; FAM, famotidine; S0859, *N*-cyanosulphonamide; CEL, celecoxib.

b MIC values were obtained from three independent biological replicates. Vancomycin and oxacillin were used as positive controls with a MIC of <2 μM for both. (EZA 64 μM = 16.5 μg/ml, 250 μM = 64.6 μg/ml).

## DISCUSSION

The discrepancies in the current literature regarding the presence of CA in S. aureus are substantial to warrant a comprehensive study to correct them, especially given that S. aureus is a clinically important pathogen. The first publication was in 1990 when Nafi et al. used a protein-binding monospecific antibody prepared against purified Neisseria sicca CA by immunoblotting method and also determined CA activity in cell extracts of various bacteria to screen for the presence of CA (18). Although CA activity was not detected in S. aureus, there was a positive reaction in the immunoblot, suggesting a reaction with a CA-like protein. In 1999, Smith et al. reported a molecular mass of 23 kDa in immunoblot with antisera raised against β-CA from Methanobacterium thermoautotrophicum ΔH and also some CA activity in S. aureus cells extract (24). Detection of target proteins by immunoreactivity alone is highly questionable in S. aureus because of its two IgG-binding proteins.

In 2015, Capasso and Supuran reported that the genome of S. aureus encodes only for γ-CA, but no other information or citation was given to support this statement (9). In the following year, the same authors stated that S. aureus has a γ-CA, referring to protein EVX10196.1, which was used to build a CA phylogenetic tree (10). In NCBI database, EVX10196.1 is annotated as 2,3,4,5-tetrahydropyridine-2,6-dicarboxylate *N*-acetyltransferase from S. aureus M20916, which is an unfinished genome with 67 contigs. This 239-amino-acid protein is listed as nonessential by AureoWiki and is annotated as *dapD*, which is part of an operon consisting of six genes involved in the biosynthesis of lysine (25). In S. aureus, lysine is an important amino acid, as it is needed not only as a building block for proteins but also as a component of the cell wall peptidoglycan. Therefore, EVX10196.1 is not a CA. Last year, the same group which reported earlier that S. aureus encodes only γ-CA now presented the production, kinetics, and inhibitory characterization of β-CA from the S. aureus (11). The CA gene was obtained from UniProt ID EZX15767 and was synthesized to produce a recombinant protein in Escherichia coli. A search in UniProt revealed that this protein is encoded by a gene V070_02709 from the S. aureus strain C0673. From our results above, C0673 is in fact S. sciuri and not S. aureus, and hence the CA activity described was actually from S. sciuri. A very recent publication from the same group followed up on the study by reporting its inhibition profile of S. aureus CA with anions and other small molecules (12). The same recombinant protein described earlier was used in this study, meaning that the CA inhibition referred to S. sciuri and not S. aureus.

Our bioinformatics analyses have clearly shown that S. aureus CAs are wrongly annotated as such, while in fact they are not present in S. aureus (Table 1 to 5). The absence of a CA in S. aureus is also supported by the deletion of *mpsABC* in four different S. aureus backgrounds (4, 17) (Fig. S1) and the fact that S. aureus is resistant to CA inhibitor EZA whereas *S. carnosus*, which has been shown to possess only CA, was inhibited by EZA (Fig. 1; Table 6; Table S6). The MIC values also imply that EZA is specific only for CA but not bicarbonate transporters (Table 6), which further complicates the treatment of pathogens such as S. aureus and S. epidermidis. The MIC values for CA-possessing *S. carnosus* and S. pseudintermedius in our study (64 to 250 μM) were comparable to those of Helicobacter pylori (also harboring CA genes), which were in the range of 200 to 300 μM for EZA. For AZA, our MIC values were >1 mM, consistent with those reported for H. pylori at 2 to 8 mM (26). In another study with vancomycin-resistant Enterococcus faecium (VRE), which harbors CA genes, the MIC values were 0.5 μM for AZA and 1 μM for EZA, while S. aureus USA300 (MRSA) showed an MIC of >16 μM for both compounds, which was the upper limit tested (20). We also tested a selective human sodium-bicarbonate cotransporter inhibitor (S0859) (Fig. S2) and found that this inhibitor has no effect on bacterial MpsAB type bicarbonate transporter in S. aureus and S. epidermidis (Table 6), suggesting distinct differences in human and bacterial bicarbonate transporters. Although these MIC data (Table 6) are preliminary and require further research, this could imply that MpsAB can be a novel and promising target for such inhibitors in the treatment of infections. Furthermore, this can also be extended to other clinically relevant pathogens, such as Bacillus anthracis, Bacillus subtilis, Legionella pneumophila, Vibrio cholerae, and Burkholderia multivorans. Based on Pfam motifs, these bacteria possess both MpsAB homologs and CAs (4), thus making them resistant to CA inhibitors.

## MATERIALS AND METHODS

### Bioinformatic analyses.

For the screening of CA based on Pfam motifs, the 259 S. aureus finished genomes in the IGM/M database (accessed 8 June 2021) were search for the presence of Pfam00484, Pfam00194, and Pfam10563 for prokaryotic-type CAs, eukaryotic-type CAs, and putative CA-like domain, respectively (Table 1). Next, the protein sequence of *S. carnosus* CA, which is a β-CA (NCBI accession number WP_015900702.1), was subjected to BLASTp (27) search in representative strains of genus Staphylococcus for the protein similarities and the presence of β-CAs (Table 1). To look for the presence of α- and γ-CAs, the sequences from some experimentally confirmed CAs as listed in Table 3 and 4 were subjected to BLASTp search in S. aureus, *S. carnosus*, and S. pseudintermedius using NCBI database (https://www.ncbi.nlm.nih.gov and http://dbis.uni-regensburg.de/frontdoor.php?titel_id=481; accessed 12 June 2021). In order to confirm that there is no CA present, the protein sequences annotated as S. aureus CA in NCBI were subjected to BLASTp search in all 259 finished and 4,590 unfinished genomes of S. aureus found in IMG/M (Table 5). Finally, these proteins annotated as S. aureus CA in NCBI and their origins were examined in NCBI Assembly database to provide the details about the strains’ numbers, assembly levels, and numbers of contigs (Table 2). Each of these protein sequences was also subjected to BLASTp search to check if it has any similarities with S. aureus protein.

### Bacteria strains and growth conditions.

All the strains used in this study are listed in Table S1. For cloning procedures, the E. coli and S. aureus strains were grown in basic medium (BM) at 37°C with shaking at 150 rpm, unless otherwise specified. The BM consists of 1% soy peptone, 0.5% yeast extract, 0.5% NaCl, 0.1% glucose, and 0.1% K_2_HPO_4_ adjusted to pH 7.2. Bacterial cultures were cultivated in 10 ml medium using baffled 100 ml flasks. When necessary, the culture medium was supplemented with the following antibiotics at the indicated concentrations: chloramphenicol at 10 μg/ml and anhydrotetracycline at 100 ng/ml for staphylococcal strains and 100 μg/ml ampicillin for E. coli strains.

### Construction of staphylococcal deletion mutants and their complementation.

The oligonucleotides used in this study are listed in Table S2. The nucleotide sequences were obtained from Kyoto Encyclopedia of Genes and Genomes (KEGG). The deletion mutant of Δ*mpsABC* in S. aureus JE2 (KEGG accession numbers SAUSA300_0425, SAUSA300_0426, and SAUSA300_0426) and MW2 (KEGG accession numbers MW0407, MW0408, and MW0409) were constructed as markerless deletions using allelic replacements as described in reference 28. Up- and downstream flanking regions were approximately 2 kb each for both deletions. The recombinant plasmid from our previous study (17) was used for transformation into S. aureus JE2, and the subsequent deletion steps were the same as those for S. aureus MW2. For the construction of S. aureus MW2 Δ*mpsABC*, the up- and downstream regions of *mpsABC* were amplified from the chromosomal DNA of S. aureus MW2. The amplified fragments were assembled using linearized plasmid pBASE6 (SmaI restriction site) (29) via Gibson assembly (30) using Hi-Fi DNA assembly master mix (New England Biolabs). The resulting plasmid was transformed into chemically competent E. coli DC10B (31). The clones harboring the right genes were then transformed into S. aureus MW2 via electroporation. Deletion of *mpsABC* in both of the strains was confirmed by PCR and sequence analysis.

Complementation of Δ*mpsABC* in both the strains was performed with the plasmid pRB473 carrying *mpsABC* along with its putative native promoter from our previous study (17). The plasmid was transformed into competent S. aureus Δ*mpsABC* JE2 and MW2, respectively, via electroporation and confirmed with PCR.

For growth visualization on agar, the wild type, Δ*mpsABC*, and its complemented mutants of S. aureus JE2 and MW2 were streaked on BM agar with inoculum adjusted to an optical density at 578 nm (OD_578_) of 0.5. The plates were incubated overnight at 37°C in atmospheric air and 5% CO_2_ conditions.

### MIC determination.

The CA inhibitors acetazolamide (AZA), ethoxyzolamide (EZA), dorzolamide (DOR), methazolamide (MEZ), celecoxib (CEL), chlorthalidone (CL), and famotidine (FAM) and selective sodium-bicarbonate cotransporters inhibitor S0859 (Table 6; Fig. S2) were purchased from Sigma-Aldrich (Germany). All the CA inhibitors were dissolved in dimethyl sulfoxide (DMSO) as stock solutions except for FAM, which was dissolved in methanol. The MIC values were determined by microdilution method according to the guidelines of Clinical and Laboratory Standards Institute (32). The CA inhibitors were serially diluted (from the highest concentration of 1 mM to the lowest concentration of 2 μM) with 50 μl of cationic adjusted Müller Hinton broth (MHB) in 96-well microtiter plates. Equal volumes of bacterial inoculum (1 × 10^6^) were added and the plates were incubated at 37°C with continuous shaking for 24 h in atmospheric air (Table 6) and, if necessary, in 5% CO_2_ conditions (Table S6). The MIC was determined as the lowest concentration that completely inhibited visible growth of the bacteria and also confirmed with a TECAN Reader (Infinite M200). Antibiotics vancomycin and oxacillin were used as standard antibiotic controls, while positive controls referred to the bacterial cells treated with DMSO or methanol at a concentration equivalent to the highest concentration used to dissolve the CA inhibitors. MHB alone was used as negative control. The MIC determinations were performed in three independent biological replicates with three technical replicates each.

For visual representation of the semiquantitative results on agar, four strains that were inhibited by EZA were used (Fig. 1). MHB agar plates were swabbed with bacterial inoculum adjusted to an OD_578_ of 0.1. Disks made of filter paper were impregnated with 10 μl of 1 mM EZA, vancomycin, and oxacillin (positive controls) and DMSO at appropriate concentration (negative control) before being placed on the agar. The agar plates were incubated overnight at 37°C in atmospheric air and 5% CO_2_ conditions.

### Data availability.

The main data supporting the findings of this work are available within the article and in the Supplemental Material or from the corresponding author upon reasonable request.

## References

[1] Casey JR. 2006. Why bicarbonate? Biochem Cell Biol 84:930–939. doi:10.1139/o06-184. 17215880

[2] Berg IA. 2011. Ecological aspects of the distribution of different autotrophic CO2 fixation pathways. Appl Environ Microbiol 77:1925–1936. doi:10.1128/AEM.02473-10. 21216907 PMC3067309

[3] Smith KS, Ferry JG. 2000. Prokaryotic carbonic anhydrases. FEMS Microbiol Rev 24:335–366. doi:10.1111/j.1574-6976.2000.tb00546.x. 10978542

[4] Fan S-H, Ebner P, Reichert S, Hertlein T, Zabel S, Lankapalli AK, Nieselt K, Ohlsen K, Götz F. 2019. MpsAB is important for *Staphylococcus aureus* virulence and growth at atmospheric CO2 levels. Nat Commun 10:3627. doi:10.1038/s41467-019-11547-5. 31399577 PMC6689103

[5] Badger M. 2003. The roles of carbonic anhydrases in photosynthetic CO(2) concentrating mechanisms. Photosynth Res 77:83–94. doi:10.1023/A:1025821717773. 16228367

[6] Mangiapia M, Usf M, Brown TW, Chaput D, Haller E, Harmer TL, Hashemy Z, Keeley R, Leonard J, Mancera P, Nicholson D, Stevens S, Wanjugi P, Zabinski T, Pan C, Scott KM, USF MCB4404L. 2017. Proteomic and mutant analysis of the CO2 concentrating mechanism of hydrothermal vent chemolithoautotroph *Thiomicrospira crunogena*. J Bacteriol 199. doi:10.1128/JB.00871-16. PMC5350277 28115547

[7] Desmarais JJ, Flamholz AI, Blikstad C, Dugan EJ, Laughlin TG, Oltrogge LM, Chen AW, Wetmore K, Diamond S, Wang JY, Savage DF. 2019. DABs are inorganic carbon pumps found throughout prokaryotic phyla. Nat Microbiol 4:2204–2215. doi:10.1038/s41564-019-0520-8. 31406332 PMC10184468

[8] Fan S-H, Matsuo M, Huang L, Tribelli PM, Friedrich G. 2021. The MpsAB bicarbonate transporter is superior to carbonic anhydrase in biofilm-forming bacteria with limited CO2 diffusion. Microbiol Spectr 9:e00305-21. doi:10.1128/Spectrum.00305-21. 34287032 PMC8552792

[9] Capasso C, Supuran CT. 2015. An overview of the alpha-, beta- and gamma-carbonic anhydrases from bacteria: can bacterial carbonic anhydrases shed new light on evolution of bacteria? J Enzyme Inhib Med Chem 30:325–332. doi:10.3109/14756366.2014.910202. 24766661

[10] Supuran CT, Capasso C. 2016. New light on bacterial carbonic anhydrases phylogeny based on the analysis of signal peptide sequences. J Enzyme Inhib Med Chem 31:1254–1260. doi:10.1080/14756366.2016.1201479. 27353388

[11] Urbanski LJ, Bua S, Angeli A, Kuuslahti M, Hytonen VP, Supuran CT, Parkkila S. 2020. Sulphonamide inhibition profile of *Staphylococcus aureus* beta-carbonic anhydrase. J Enzyme Inhib Med Chem 35:1834–1839. doi:10.1080/14756366.2020.1826942. 32972256 PMC7534311

[12] Urbanski LJ, Vullo D, Parkkila S, Supuran CT. 2021. An anion and small molecule inhibition study of the beta-carbonic anhydrase from *Staphylococcus aureus*. J Enzyme Inhib Med Chem 36:1088–1092. doi:10.1080/14756366.2021.1931863. 34056990 PMC8168783

[13] Chen IA, Chu K, Palaniappan K, Ratner A, Huang J, Huntemann M, Hajek P, Ritter S, Varghese N, Seshadri R, Roux S, Woyke T, Eloe-Fadrosh EA, Ivanova NN, Kyrpides NC. 2021. The IMG/M data management and analysis system v.6.0: new tools and advanced capabilities. Nucleic Acids Res 49:D751–D763. doi:10.1093/nar/gkaa939. 33119741 PMC7778900

[14] Fuchs S, Mehlan H, Bernhardt J, Hennig A, Michalik S, Surmann K, Pane-Farre J, Giese A, Weiss S, Backert L, Herbig A, Nieselt K, Hecker M, Volker U, Mäder U. 2018. AureoWiki-the repository of the *Staphylococcus aureus* research and annotation community. Int J Med Microbiol 308:558–568. doi:10.1016/j.ijmm.2017.11.011. 29198880

[15] Jolley KA, Bray JE, Maiden MCJ. 2018. Open-access bacterial population genomics: BIGSdb software, the PubMLST.org website and their applications. Wellcome Open Res 3:124. doi:10.12688/wellcomeopenres.14826.1. 30345391 PMC6192448

[16] Smits THM. 2019. The importance of genome sequence quality to microbial comparative genomics. BMC Genomics 20:662. doi:10.1186/s12864-019-6014-5. 31429698 PMC6701015

[17] Mayer S, Steffen W, Steuber J, Götz F. 2015. The *Staphylococcus aureus* NuoL-like protein MpsA contributes to the generation of membrane potential. J Bacteriol 197:794–806. doi:10.1128/JB.02127-14. 25448817 PMC4325100

[18] Nafi BM, Miles RJ, Butler LO, Carter ND, Kelly C, Jeffery S. 1990. Expression of carbonic anhydrase in neisseriae and other heterotrophic bacteria. J Med Microbiol 32:1–7. doi:10.1099/00222615-32-1-1. 2111405

[19] Abutaleb NS, Elkashif A, Flaherty DP, Seleem MN. 2021. In vivo antibacterial activity of acetazolamide. Antimicrob Agents Chemother 65. doi:10.1128/AAC.01715-20. PMC8097480 33495225

[20] Younis W, AbdelKhalek A, Mayhoub AS, Seleem MN. 2017. In vitro screening of an FDA-approved library against ESKAPE pathogens. Curr Pharm Des 23:2147–2157. doi:10.2174/1381612823666170209154745. 28190396 PMC5662795

[21] Swenson ER. 2014. Safety of carbonic anhydrase inhibitors. Expert Opin Drug Saf 13:459–472. doi:10.1517/14740338.2014.897328. 24611470

[22] Howard JM, Chremos AN, Collen MJ, Mcarthur KE, Cherner JA, Maton PN, Ciarleglio CA, Cornelius MJ, Gardner JD, Jensen RT. 1985. Famotidine, a new, potent, long-acting histamine H2-receptor antagonist - comparison with cimetidine and ranitidine in the treatment of Zollinger-Ellison syndrome. Gastroenterology 88:1026–1033. doi:10.1016/s0016-5085(85)80024-x. 2857672

[23] Ch’en FF, Villafuerte FC, Swietach P, Cobden PM, Vaughan-Jones RD. 2008. S0859, an N-cyanosulphonamide inhibitor of sodium-bicarbonate cotransport in the heart. Br J Pharmacol 153:972–982. doi:10.1038/sj.bjp.0707667. 18204485 PMC2267275

[24] Smith KS, Jakubzick C, Whittam TS, Ferry JG. 1999. Carbonic anhydrase is an ancient enzyme widespread in prokaryotes. Proc Natl Acad Sci USA 96:15184–15189. doi:10.1073/pnas.96.26.15184. 10611359 PMC24794

[25] Wiltshire MD, Foster SJ. 2001. Identification and analysis of *Staphylococcus aureus* components expressed by a model system of growth in serum. Infect Immun 69:5198–5202. doi:10.1128/IAI.69.8.5198-5202.2001. 11447207 PMC98621

[26] Modak JK, Tikhomirova A, Gorrell RJ, Rahman MM, Kotsanas D, Korman TM, Garcia-Bustos J, Kwok T, Ferrero RL, Supuran CT, Roujeinikova A. 2019. Anti-*Helicobacter pylori* activity of ethoxzolamide. J Enzyme Inhib Med Chem 34:1660–1667. doi:10.1080/14756366.2019.1663416. 31530039 PMC6759998

[27] Boratyn GM, Camacho C, Cooper PS, Coulouris G, Fong A, Ma N, Madden TL, Matten WT, McGinnis SD, Merezhuk Y, Raytselis Y, Sayers EW, Tao T, Ye J, Zaretskaya I. 2013. BLAST: a more efficient report with usability improvements. Nucleic Acids Res 41:W29–W33. doi:10.1093/nar/gkt282. 23609542 PMC3692093

[28] Bae T, Schneewind O. 2006. Allelic replacement in *Staphylococcus aureus* with inducible counter-selection. Plasmid 55:58–63. doi:10.1016/j.plasmid.2005.05.005. 16051359

[29] Geiger T, Francois P, Liebeke M, Fraunholz M, Goerke C, Krismer B, Schrenzel J, Lalk M, Wolz C. 2012. The stringent response of *Staphylococcus aureus* and its impact on survival after phagocytosis through the induction of intracellular PSMs expression. PLoS Pathog 8:e1003016. doi:10.1371/journal.ppat.1003016. 23209405 PMC3510239

[30] Gibson DG, Young L, Chuang RY, Venter JC, Hutchison CA, 3rd, Smith HO. 2009. Enzymatic assembly of DNA molecules up to several hundred kilobases. Nat Methods 6:343–345. doi:10.1038/nmeth.1318. 19363495

[31] Monk IR, Shah IM, Xu M, Tan MW, Foster TJ. 2012. Transforming the untransformable: application of direct transformation to manipulate genetically *Staphylococcus aureus* and *Staphylococcus epidermidis*. mBio 3. doi:10.1128/mBio.00277-11. PMC3312211 22434850

[32] Cockerill FR, Clinical and Laboratory Standards Institute. 2012. Methods for dilution antimicrobial susceptibility tests for bacteria that grow aerobically approved standard, 9th ed. CLSI, Wayne, PA.

[33] Kaur J, Cao X, Abutaleb NS, Elkashif A, Graboski AL, Krabill AD, AbdelKhalek AH, An W, Bhardwaj A, Seleem MN, Flaherty DP. 2020. Optimization of acetazolamide-based scaffold as potent inhibitors of vancomycin-resistant Enterococcus. J Med Chem 63:9540–9562. doi:10.1021/acs.jmedchem.0c00734. 32787141 PMC8317130

[34] Chirica LC, Elleby B, Lindskog S. 2001. Cloning, expression and some properties of alpha-carbonic anhydrase from *Helicobacter pylori*. Biochim Biophys Acta 1544:55–63. doi:10.1016/s0167-4838(00)00204-1. 11341916

[35] Chirică LC, Elleby B, Jonsson BH, Lindskog S. 1997. The complete sequence, expression in *Escherichia col*i, purification and some properties of carbonic anhydrase from *Neisseria gonorrhoeae*. Eur J Biochem 244:755–760. doi:10.1111/j.1432-1033.1997.00755.x. 9108244

[36] Elleby B, Chirica LC, Tu C, Zeppezauer M, Lindskog S. 2001. Characterization of carbonic anhydrase from *Neisseria gonorrhoeae*. Eur J Biochem 268:1613–1619. doi:10.1046/j.1432-1327.2001.02031.x. 11248679

[37] Del Prete S, Isik S, Vullo D, De Luca V, Carginale V, Scozzafava A, Supuran CT, Capasso C. 2012. DNA cloning, characterization, and inhibition studies of an alpha-carbonic anhydrase from the pathogenic bacterium *Vibrio cholerae*. J Med Chem 55:10742–10748. doi:10.1021/jm301611m. 23181552

[38] Keilin D, Mann T. 1940. Carbonic anhydrase. Purification and nature of the enzyme. Biochem J 34:1163–1176. doi:10.1042/bj0341163. 16747299 PMC1265396

[39] Lowe N, Edwards YH, Edwards M, Butterworth PH. 1991. Physical mapping of the human carbonic anhydrase gene cluster on chromosome 8. Genomics 10:882–888. doi:10.1016/0888-7543(91)90176-f. 1916821

[40] Murakami H, Marelich GP, Grubb JH, Kyle JW, Sly WS. 1987. Cloning, expression, and sequence homologies of cDNA for human carbonic anhydrase II. Genomics 1:159–166. doi:10.1016/0888-7543(87)90008-5. 3121496

[41] Merlin C, Masters M, McAteer S, Coulson A. 2003. Why is carbonic anhydrase essential to *Escherichia coli*? J Bacteriol 185:6415–6424. doi:10.1128/JB.185.21.6415-6424.2003. 14563877 PMC219403

[42] Park HM, Park JH, Choi JW, Lee J, Kim BY, Jung CH, Kim JS. 2012. Structures of the gamma-class carbonic anhydrase homologue YrdA suggest a possible allosteric switch. Acta Crystallogr D Biol Crystallogr 68:920–926. doi:10.1107/S0907444912017210. 22868757

[43] Alber BE, Ferry JG. 1994. A carbonic anhydrase from the archaeon *Methanosarcina thermophila*. Proc Natl Acad Sci USA 91:6909–6913. doi:10.1073/pnas.91.15.6909. 8041719 PMC44307

[44] Vogler M, Karan R, Renn D, Vancea A, Vielberg MT, Grotzinger SW, DasSarma P, DasSarma S, Eppinger J, Groll M, Rueping M. 2020. Crystal structure and active site engineering of a halophilic gamma-carbonic anhydrase. Front Microbiol 11:742. doi:10.3389/fmicb.2020.00742. 32411108 PMC7199487

