# Supplementary material for: Staphylococcus aureus Genomes Harbor Only MpsAB-Like Bicarbonate Transporter but Not Carbonic Anhydrase as Dissolved Inorganic Carbon Supply System
Source: Microbiol Spectr. 2021 Nov 3;9(3):e00970-21. doi: 10.1128/Spectrum.00970-21 (PMC8567241; doi:10.1128/Spectrum.00970-21)
Supplement: SUPPLEMENTAL FILE 1 — Supplemental material. Download SPECTRUM00970-21_Supp_1_seq4.pdf, PDF file, 0.4 MB [file spectrum00970-21_supp_1_seq4.pdf]

SUPPLEMENTARY MATERIALS

Staphylococcus aureus **genomes harbor only MpsAB-like bicarbonate transporter but not carbonic anhydrase as dissolved inorganic carbon supply system**

Sook-Ha Fan, Elisa Liberini, and Friedrich Götz [#]

Supplementary Figures

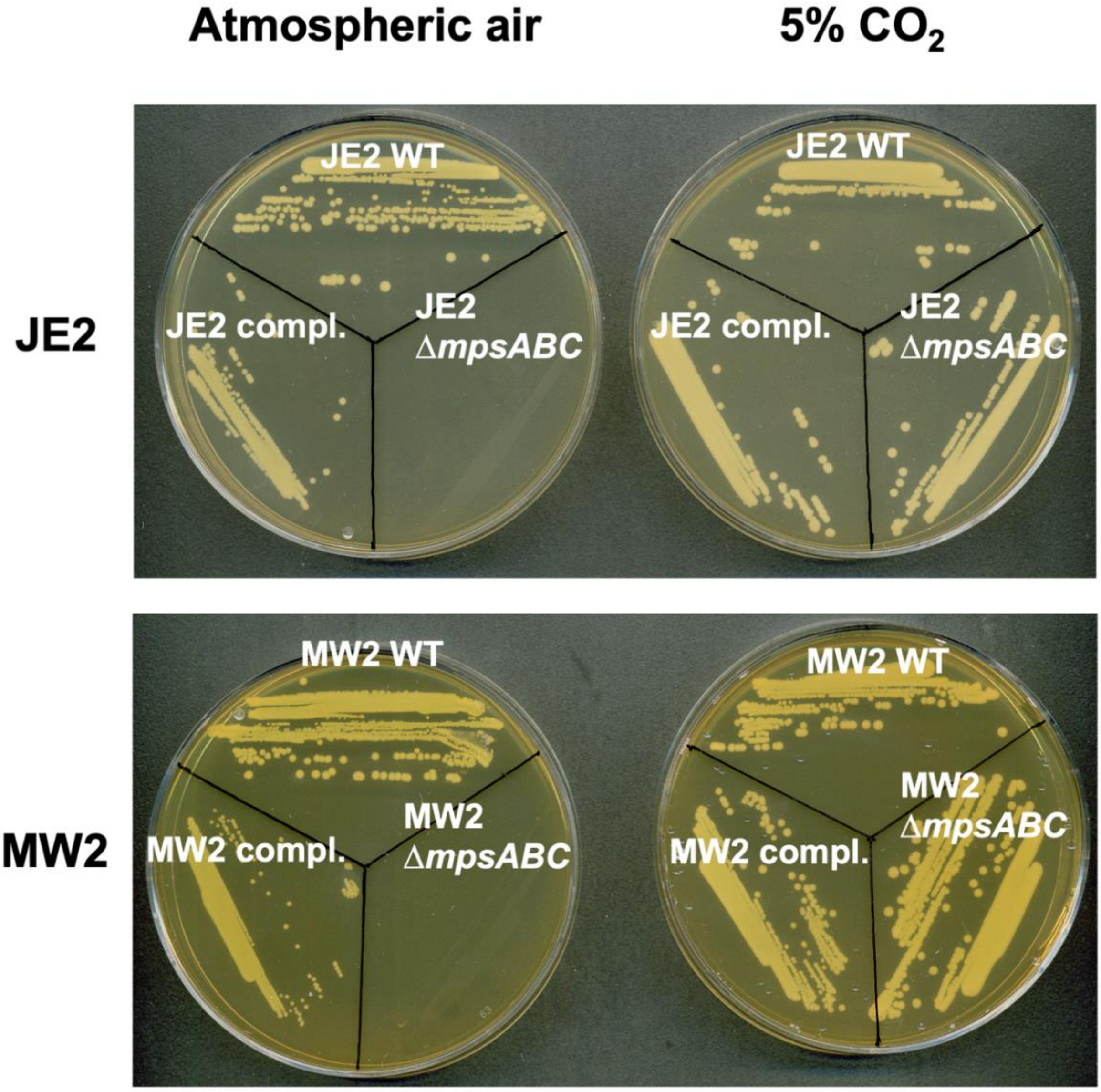

Supplementary Figure 1: Deletion of *mpsABC* in MRSA strains *S. aureus* JE2 (top) and MW2 (below) grown in atmospheric air (left) and 5% CO_2 (right). Clockwise from top: Wild type (WT); deletion of *mpsABC* (Δ*mpsABC*) and complemented mutant (Δ*mpsABC* pRB473*mpsABC*).

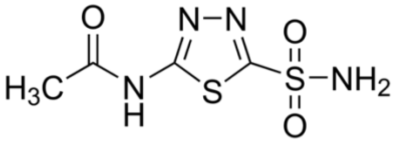

Acetazolamide (AZA)
MW: 222.25

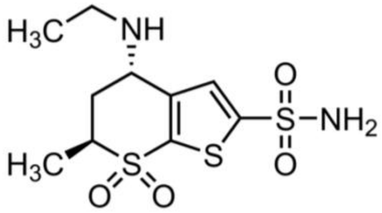

Dorzolamide (DOR)
MW: 324.44

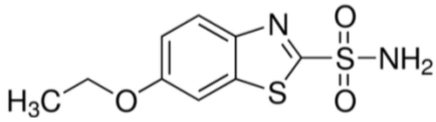

Ethoxyzolamide (EZA)
MW: 258.32

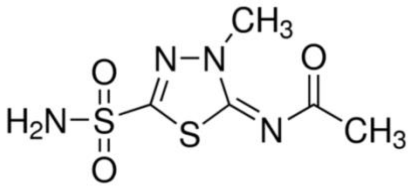

Methazolamide (MEZ)
MW: 236.27

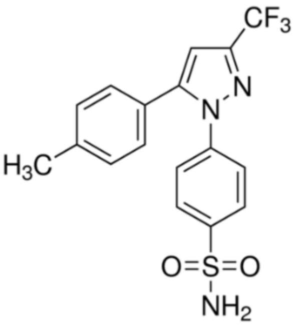

Celecoxib
MW: 318.37

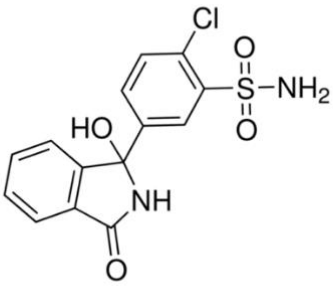

Chlorthalidone (CL)
MW: 338.77

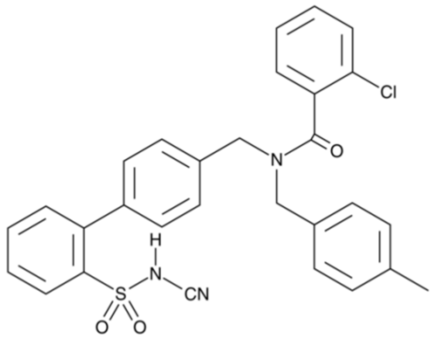

S0859 (N-cyanosulphonamide)
MW: 530.04

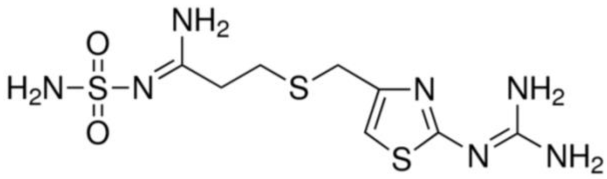

Famotidine
MW: 337.45

Supplementary Figure 2: Chemical structures of the carbonic anhydrase inhibitors and other compounds used in the MIC determinations. Ethoxyzolamide (EZA) (highlighted in red) was the only compound showing MIC values against CA-harboring strains *S. carnosus* and *S. pseudintermedius*. All images were obtained from Sigma-Aldrich's website except for S0859 which was from Cayman Chemical. (MW: Molecular weight)

Supplementary Table 1: Bacterial strains used in this study

Strain	Description	Ref.
S. aureus HG001	*S. aureus* NCTC8325 derivative with repaired *rsbU*	(1)
S. aureus HG001 Δ*mpsABC* (pRB473 *can*)	*S. aureus* HG001Δ*mpsABC* complemented with CA from *S. carnosus* TM300	(2)
S. aureus HG001 (pRB473 *can*)	*S. aureus* HG001 (pRB473- *can*) carrying CA from *S. carnosus* TM300 (having both Dissolved Inorganic Carbon Supply [DICS] system)	(2)
S. aureus JE2	*S. aureus* USA 300 FPR375 derivative, cured of three plasmids	(3)
S. aureus JE2 Δ*mpsABC*	*S. aureus* JE2 Δ*mpsABC* (deletion of SAUSA300_0425, SAUSA300_0426 and SAUSA300_0426)	This study
S. aureus JE2 Δ*mpsABC* (pRB473 *mpsABC*)	*S. aureus* JE2 Δ*mpsABC* complemented with *mpsABC*	This study
S. aureus MW2	Community-associated methicillin resistant clinical isolate, pulsed- field type USA400	(4)
S. aureus MW2 Δ*mpsABC*	*S. aureus* MW2 Δ*mpsABC* (deletion of MW0407, MW0408 and MW0409)	This study

S. aureus MW2 Δ*mpsABC* (pRB473 *mpsABC*)	*S. aureus* MW2 Δ*mpsABC* complemented with *mpsABC*	This study
S. carnosus TM300	Food grade staphylococcal strain lacking genes involved in pathogenicity	(5)
S. carnosus TM300 Δ*can* (pRB473 *mpsABC*)	*S. carnosus* TM300Δ*can* complemented with *mpsABC* from *S. aureus*	(2)
S. carnosus TM300 (pRB473 *mpsABC*)	*S. carnosus* TM300 carrying *mpsABC* from *S. aureus* HG001 (having both Dissolved Inorganic Carbon Supply [DICS] system)	(2)
S. carnosus TM300 (pRB473 *can*)	*S. carnosus* TM300 carrying a plasmid harboring an extra CA gene (overexpression of CA)	This study
S. epidermidis O47	Clinical isolate from patient with orthopedic implant infection	(6)
S. pseudintermedius ED99	Clinical isolate of canine pathogen	(7)
S. pseudintermedius ED99 Δ*can* (pRB473 *mpsABC*)	*S. pseudintermedius* ED99Δ*can* complemented with *mpsABC* from *S. aureus*	(2)

Supplementary Table 2: Oligonucleotides used in this study

**Primer name**	**Sequence (5'→3')**
For the construction of *S. aureus* JE2 Δ*mpsABC* [recombinant plasmid from (8)]	
1_attB1_412	GGGGACAAGTTTGTACAAAAAAGCAGGCTCCATGCTAAAAGGTTCAATGATTGG
2_412-414_SacII	TATATCCGCGGCTAATCTCTCGCATAATTGCTTATG
3_412-414_SacII	TATATCCGCGGCTTGAGGATAATTTGGAAAAGC
4_attB2_412-414	GGGGACCACTTTGTACAAGAAAGCTGGGTGTGCACTTGTTTCATAGGATGGCGC
For the construction of *S. aureus* MW2 Δ*mpsABC*	
MW2 up mpsABC_fwd	AATTCCGGAGCTCGGTACCCTCCATGCTAAAAGGTTCAAT
MW2 up mpsABC rev	TATCCTCAAGCTAATCTCTCGCATAATTGC
MW2 down mpsABC fwd	GAGAGATTAGCTTGAGGATAATTTGGAAAAG
MW2 down mpsABC rev	ACAGATCTGCGCGCTAGCCCTGTGCACTTGTTTCATAG

Supplementary Table 3: MIC values of Ethoxyzolamide (EZA) against selected staphylococcal strains in atmospheric air and CO_2 conditions.

**Dissolved Inorganic Carbon Supply (DICS) system**	**Strain**	**MIC (µM)***			
		**EZA**		**VAN/OXA**	
		**A**	**5% CO_2**	**A**	**5% CO_2**
**MpsAB**	*S. aureus* HG001	> 1000	> 1000	< 2	< 2
**CA**	*S. carnosus* TM300	64	> 1000	< 2	< 2
	S. carnosus TM300 (pRB473 *can*)	250	> 1000	< 2	< 2
	S. pseudintermedius ED99	250	>1000	< 2	< 2

EZA: Ethoxyzolamide; **VAN/OXA**: Vancomycin/Oxacillin; **A**: Atmospheric air conditions. * MIC values were obtained from three independent biological replicates. (EZA 64 µM = 16.5 µg/ml, 250 µM = 64.6 µg/ml)

Supplementary References

1. Herbert S, Ziebandt AK, Ohlsen K, Schafer T, Hecker M, Albrecht D, Novick R, Götz F. 2010. Repair of global regulators in *Staphylococcus aureus* 8325 and comparative analysis with other clinical isolates. Infect Immun 78:2877-2889.
2. Fan S-H, Matsuo M, Huang L, Tribelli PM, F. G. 2021. The MpsAB bicarbonate transporter is superior to carbonic anhydrase in biofilm-forming bacteria with limited CO2 diffusion. Microbiol Spectr 2:e00305-21.
3. Fey PD, Endres JL, Yajjala VK, Widhelm TJ, Boissy RJ, Bose JL, Bayles KW. 2013. A genetic resource for rapid and comprehensive phenotype screening of nonessential Staphylococcus aureus genes. mBio 4:e00537-12.
4. Baba T, Takeuchi F, Kuroda M, Yuzawa H, Aoki K, Oguchi A, Nagai Y, Iwama N, Asano K, Naimi T, Kuroda H, Cui L, Yamamoto K, Hiramatsu K. 2002. Genome and virulence determinants of high virulence community-acquired MRSA. Lancet 359:1819-27.
5. Wagner E, Doskar J, Gotz F. 1998. Physical and genetic map of the genome of *Staphylococcus carnosus* TM300. Microbiology (Reading) 144 (Pt 2):509-17.
6. Raue S, Fan SH, Rosenstein R, Zabel S, Luqman A, Nieselt K, Gotz F. 2020. The Genome of *Staphylococcus epidermidis* O47. Front Microbiol 11:2061.
7. Ben Zakour NL, Bannoehr J, van den Broek AH, Thoday KL, Fitzgerald JR. 2011. Complete genome sequence of the canine pathogen *Staphylococcus pseudintermedius*. J Bacteriol 193:2363-4.
8. Mayer S, Steffen W, Steuber J, Götz F. 2015. The *Staphylococcus aureus* NuoL-Like Protein MpsA Contributes to the Generation of Membrane Potential. J Bacteriol 197:794-806.
